# Exploring the Biochemical Origin of DNA Sequence Variation in Barley Plants Regenerated via in Vitro Anther Culture

**DOI:** 10.3390/ijms21165770

**Published:** 2020-08-11

**Authors:** Piotr T. Bednarek, Jacek Zebrowski, Renata Orłowska

**Affiliations:** 1 Department of Plant Physiology and Biochemistry, Plant Breeding and Acclimatization Institute—National Research Institute, Radzików, 05-870 Błonie, Poland; r.orlowska@ihar.edu.pl; 2 Institute of Biology and Biotechnology, University of Rzeszow, Al. Rejtana 16c A, 35-959 Rzeszow, Poland; jaze28@interia.pl

**Keywords:** ATR-FTIR spectroscopy, β-glucans, cellulose, DNA demethylation, S-adenosyl L-methionine, sequence variation, time of in vitro culture

## Abstract

Tissue culture is an essential tool for the regeneration of uniform plant material. However, tissue culture conditions can be a source of abiotic stress for plants, leading to changes in the DNA sequence and methylation patterns. Despite the growing evidence on biochemical processes affected by abiotic stresses, how these altered biochemical processes affect DNA sequence and methylation patterns remains largely unknown. In this study, the methylation-sensitive Amplified Fragment Length Polymorphism (metAFLP) approach was used to investigate de novo methylation, demethylation, and sequence variation in barley regenerants derived by anther culture. Additionally, we used Attenuated Total Reflectance Fourier Transform Infrared (ATR-FTIR) spectroscopy to identify the spectral features of regenerants, which were then analyzed by mediation analysis. The infrared spectrum ranges (710–690 and 1010–940 cm^−1^) identified as significant in the mediation analysis were most likely related to β-glucans, cellulose, and S-adenosyl-L-methionine (SAM). Additionally, the identified compounds participated as predictors in moderated mediation analysis, explaining the role of demethylation of CHG sites (CHG_DMV) in in vitro tissue culture-induced sequence variation, depending on the duration of tissue culture. The data demonstrate that ATR-FTIR spectroscopy is a useful tool for studying the biochemical compounds that may affect DNA methylation patterns and sequence variation, if combined with quantitative characteristics determined using metAFLP molecular markers and mediation analysis. The role of β-glucans, cellulose, and SAM in DNA methylation, and in cell wall, mitochondria, and signaling, are discussed to highlight the putative cellular mechanisms involved in sequence variation.

## 1. Introduction

It is commonly believed that sequence variation during in vitro tissue culture is either caused by the activation of transposons [1], resulting in DNA demethylation [2] during cell reprogramming [3], or by the presence of modified cytosines [4], which undergo further modifications [5] but escape DNA repair mechanisms [6]. DNA demethylation is a widely used epigenetic marker in plants. DNA demethylation occurs in two sequence contexts: symmetric and asymmetric [7]. Symmetric DNA demethylation affects CG and CHG sequences (where H = A, T, and C) predominantly in gene-rich euchromatic regions, whereas asymmetric DNA demethylation affects CHH sequences mostly in heterochromatic regions. The maintenance of both types of DNA methylation patterns is regulated by distinct mechanisms [7].

Two mechanisms of DNA demethylation have been identified: passive and active. The passive mechanism assumes that DNA loses epigenetic marks during replication in the absence of the methylation of newly synthesized DNA strands by DNA (cytosine-5)-methyltransferase 1 (DNMT1), an enzyme that catalyzes the transfer of methyl groups to specific CpG sites during replication [8]. DNMT1 is responsible for the maintenance of DNA methylation patterns during replication. Passive demethylation has been reported in the male gametophyte and endosperm with reduced RNA-directed DNA methylation (RdDM) factors [9].

Active DNA demethylation utilizes 5-methylcytosine (5 mC) DNA glycosylases [10] and is a distinct feature of the maternal central cell. DEMETER-LIKE 2 (DML2) and DML3 function in nonsomatic and somatic tissues [11], and delete the methylated cytosines via the base excision repair (BER) pathway [12,13]. Active DNA demethylation depends on the REPRESSOR OF SILENCING 1 (ROS1) family, which comprises of 5 mC DNA glycosylases and apurinic/apyrimidinic lyases that antagonize de novo DNA methylation via the RdDM pathway to prevent DNA hypermethylation. ROS1 counteracts de novo DNA methylation in transgenes by actively promoting DNA demethylation. Other enzymes such as DEMETER (DME), DML2, and DML3 also participate in this process. DME regulates gene imprinting [14,15], whereas DML2 and DML3 are involved in the proper distribution of DNA methylation marks [13]. Recent studies show that active DNA demethylation is mediated by the increased DNA methylation (IDM) complex. Moreover, de novo DNA methylation and demethylation are under the control of a methylation-sensing genetic element [8]. In *Arabidopsis thaliana*, both de novo DNA methylation and demethylation processes are required for cellular reprogramming during in vitro tissue culture [16].

In active DNA demethylation, DNA glycosylases remove 5 mC and cleave the N-glycosidic (base–sugar) bond, generating apurinic or apyrimidinic sites, followed by gap-filling by a DNA polymerase and a DNA ligase [17]. In addition to the BER pathway, DNA demethylation is also accomplished by the removal of a DNA fragment, and the gap is filled using new nucleotides via the nucleotide excision (NER) or mismatch repair (MMR) mechanism. The NER and MMR mechanisms rely on the removal of 5 mC by further modifications such as oxidation of the methyl group, leading to the formation of 5-hydroxymethylcytosine (5 hmC) [5,18]. In active DNA demethylation, deamination of 5 mC generates thymine (T), which may result in C→T transition, as T is not recognized by the uracil-DNA glycosylase. Thus, mutations due to deamination are the most frequent, as they escape the cell repair system. Alternatively, oxidation of 5 mC may also contribute to sequence variation [19].

Recent studies demonstrate that abiotic stress conditions are responsible for DNA methylation-dependent stress memory in plants [20]. In some cases, changes in DNA methylation patterns are associated with plant stress responses [21]. Furthermore, abiotic stresses such as cold and drought are responsible for changes in DNA methylation patterns [22]. Additionally, studies on plant responses to in vitro tissue culture-induced stress show that tissue culture conditions promote changes in stress-induced DNA methylation and/or demethylation patterns [23,24,25,26,27]. Machczyńska and colleagues reported a relatively high level of DNA sequence variation between the donor plant and its regenerants [28]. However, evidence supporting a direct link between DNA demethylation/sequence variation and in vitro tissue culture conditions is lacking.

Sequence variation induced by in vitro tissue culture has been investigated using the methylation-sensitive Amplified Fragment Length Polymorphism (metAFLP) approach [28,29], which provides quantitative information about the DNA sequence. Furthermore, Attenuated Total Reflectance Fourier Transform Infrared (ATR-FTIR) spectroscopy has been used to analyze the extent to which nucleotide variation affects biochemical compounds in plants regenerated by an in vitro culture [30]. ATR-FTIR spectroscopy is a powerful technique that has been widely used to analyze the biochemical profiles of diverse biological materials, including plant tissues [31,32]. ATR-FTIR spectroscopy is a fast and simple measurement procedure that does not require special sample preparation, thus allowing the examination of a large number of samples. Although ATR-FTIR spectroscopy is not similarly precise in quantitative analysis of a specific analyte like time-consuming chromatography, it does allow for comprehensive survey of all essential compounds present in the material under investigation on the basis of a single short-time measurement. ATR-FTIR spectroscopy is generally used in combination with various mathematical and statistical tools that enhance its research potential, e.g., to discriminate between samples according to taxonomic categories [33,34] or to determine susceptibility to stresses [35,36,37]. Additionally, mediation analysis is often used as a method of choice to study the relationship between distinct compounds [38] as it does not involve expensive molecular experiments and may support valuable data on biological mechanisms.

Previously, we showed that in vitro tissue culture is moderated by copper (Cu^2+^) and silver (Ag^+^) ions present in the regeneration medium [38], depending on the duration of culture. Mediation analysis [38] showed that sequence variation in barley plants obtained by tissue culture was due to the presence of Cu^2+^ and Ag^+^ ions affecting CG sequences in different contexts, depending on the duration of tissue culture. Therefore, we hypothesized that sequence variation within other contexts might be induced by certain cellular compounds under the influence of abiotic stress, and this sequence variation may occur either throughout the duration of tissue culture or preferentially during specific intervals. To test this hypothesis, we investigated the putative origin of in vitro anther culture-induced sequence variation in barley regenerants using metAFLP markers, ATR-FTIR spectroscopy, and mediation analysis.

## 2. Results

Another culture of barley was performed in vitro under nine distinct treatments (M1–M9). Each treatment was conducted for a different duration and contained a different concentration of Cu^2+^ and Ag^+^ ions. A total of 35 barley regenerants were obtained. No morphological differences were detected among the regenerants, and all regenerants were in the type of an anther tissue donor plant. DNA isolated from fresh leaves of a donor and regenerated DH plants using a commercial kit yielded intact samples without detectable impurities.

Genotyping of the isolated DNAs using the metAFLP approach allowed the identification of 407 markers shared by the donor plant and regenerants [25]. Quantitative metAFLP characteristics of the DNAs are listed in Table 1.

### 2.1. ATR-FTIR Spectroscopy

**Figure 1:**
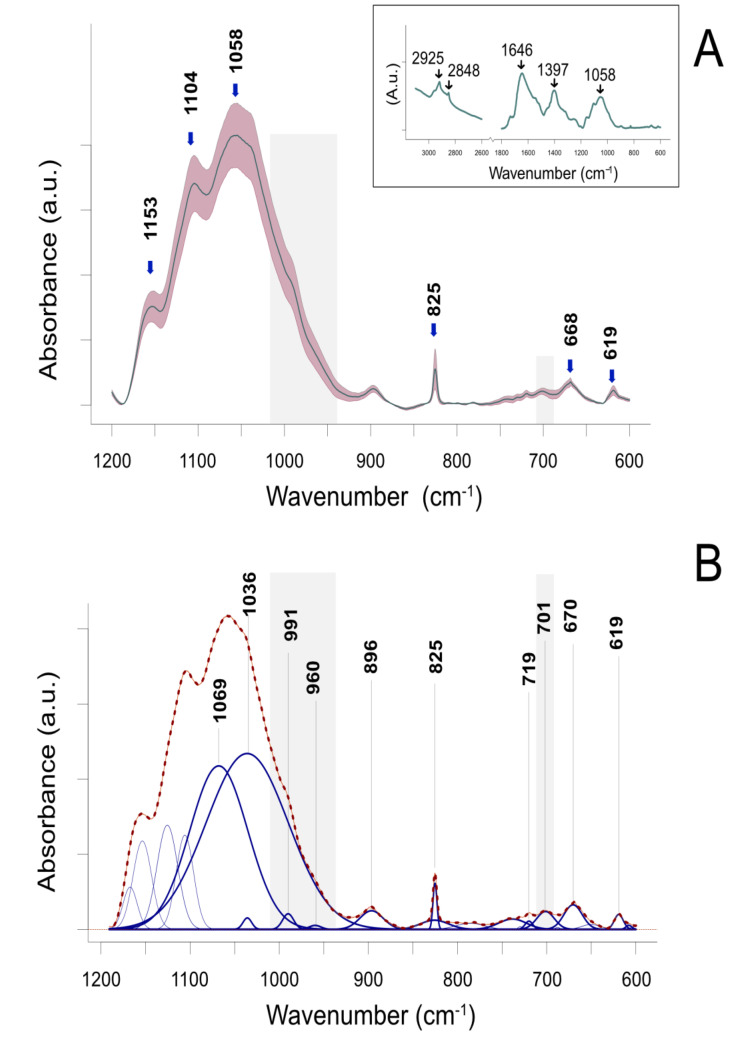
Attenuated Total Reflectance Fourier Transform Infrared (ATR-FTIR) spectrum of the leaves of barley plants regenerated by an in vitro anther culture. (**A**) ATR-FTIR spectra of the region between 1200 and 600 cm^−1^. The most prominent peaks are marked. The inset presents an extended range of the spectrum between 3100 and 600 cm^−1^. Data represent mean ± standard deviation (SD). (**B**) Numerical (Gaussian) deconvolution of overlapping peaks. Data represent the sum of fitted curves (dashed line) closely overlapping the experimental data (solid red line). The centered frequencies of major resolved peaks are shown in blue. Regions shaded in gray represent spectral regions relevant to mediation analysis.

ATR-FTIR spectroscopy was carried out to investigate the major biochemical features of the leaves of barley regenerants in a fast and straightforward manner. Figure 1A summarizes the variability in infrared data between all treatments. The spectra showed typical features of plants (Figure 1A, inset), with several usually complex broad peaks. Bands representing asymmetric and symmetric methylene (CH_2_) stretching vibrations were located at 2925 and 2848 cm^−1^, respectively, while the amide I band was centered at 1646 cm^−1^. In turn, the fingerprint region was characterized by two dominant peaks: one at 1397 cm^−1^, putatively assigned to the symmetric stretching vibrations of COO^−^, CH_2_ scissor, and CH bending [39,40], and the other at 1058 cm^−1^, assigned to the C-O, C-O-C stretch, and ring vibrations, attributed mainly to polysaccharides [41,42]. The spectral range between 1200 and 600 cm^−1^, which was the focus of the present study, is depicted in Figure 1A. The local peak at 1153 cm^−1^ was assigned mostly to C-O-C asymmetric stretch vibrations, which are characteristic of various cell wall polysaccharides and therefore are not distinctive. The local peak at 1104 cm^−1^ representing C-O, C-O-C stretch, and ring vibrations is attributed mostly to cellulose [42,43]. To resolve the broad complex band centered at 1058 cm^−1^, we performed Gaussian deconvolution of the wavenumber range going from 1200 to 600 cm^−1^, achieving a very close fit to the original contour. The component peaks are presented in Figure 1B. The band at 1069 cm^−1^ was assigned mostly to C–O and C–C stretching vibrations of β-(1→3, 1→4) glucan [44], whereas the band at 1036 cm^−1^ was attributed to the C-O-C skeletal vibration of the polysaccharide ring of both cellulose and β-glucan [42]. Two small peaks were also generated at 991 and 960 cm^−1^, which seemed to contribute to the shape of the band shoulder; the former peak putatively reflects the β-glycosidic linkage, whereas the latter peak does not seem to be related to cell wall polysaccharides. The band at 960 cm^−1^ was attributed to C–O and C–C stretching in deoxyribose [45].

The band at 896 cm^−1^ was assigned to β-glycosidic (C1-H) deformation [42]. A narrow, moderately intense peak at 825 cm^−1^ was probably caused by phenolic compounds containing a specifically substituted benzene ring; however, this peak was not considered essential in the present study. The resolved peak in the low-frequency region showed compounds at 719, 710, and 670 cm^−1^ [46]. Two bands at 701 and 670 cm^−1^ typically represent, in the case of cell wall material, H-bonded O-H out-of-plane bending vibrations of cellulose [47,48]. However, these bands may be associated with vibrations unrelated to cell wall compounds, e.g., C-S stretching vibrations present in thiols or other compounds with C-S-C bonds [49,50,51].

### 2.2. Mediation Analysis

The analysis consisted of a series of regression mediation analyses that aimed to establish whether the DNA demethylation affecting CHG contexts (CHG_DMV) was a mediator in the relationship among F710.690, F1010.940, and F710.690-F1010.940 variables and sequence variation (SV). We have also tested the model of relationships between F710.690-F1010.940 and SV mediated by CHG_DMV and moderated by the time of in vitro tissue cultures.

**Figure 2:**
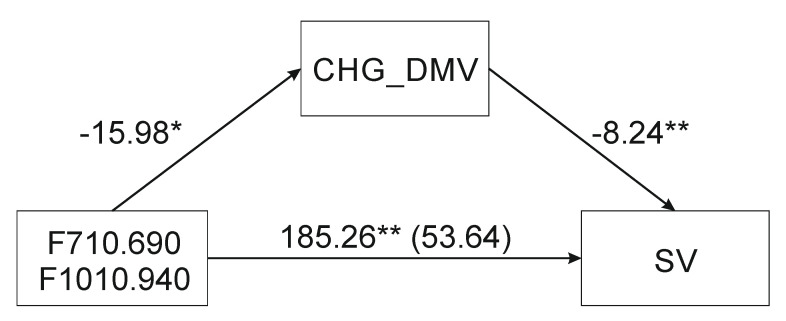
Mediation analysis of compounds associated with infrared spectral regions of 710–690 and 1010–940 cm^−1^ to demonstrate their effect on sequence variation (SV) via the demethylation of the CHG sequence context (CHG_DMV). * *p* < 0.05, ** *p* < 0.01

The F710.690 mediation on sequence variation through CHG_DMV (mediator) was assumed to be insignificant based on indirect and partial indirect effects (not shown). Based on the complete standardized indirect effect, the mediation was nearly significant (Table 2). Moreover, based on the casual mediation approach [52] coefficient relating F710.690 and SV (path c) was significant, coefficient relating F710.690 and CHG_DMV (*a*) was significant. Furthermore, the CHG_DMV (mediator) was related to the SV (DV) when both F710.690 and CHG_DMV are predictors of the dependent variable (*c’*), the coefficient relating the F710.690 to the SV (*c*) was larger (in absolute value) than the coefficient relating the F710.690 to the SV in the regression model with both the F710.690 and the CHG_DMV (*c’*) predicting the dependent variable and indicating mediation. There were significant mediation effects of F1010.940 and F710.690-F1010.940 (Figure 2) as independent variables (IDs) on sequence variation through CHG_DMV (mediator). Table 2 shows the outcomes of simple mediation analyses from IDs to SV assessing the indirect effects of CHG_DMV (indicated by a 95% confidence interval). All indirect effects except for the first model were positive. Mediation analysis showed that F1010.940 ATR-FTIR spectrum was a predictor of mediation, based on the indirect effect (IE) bootstrap value. Similarly, the indirect effect of the F710.690-F1010.940 variable on independent SV variables via the CHG_DMV (mediator) was significantly different from zero. The significance of mediations was assessed based on the Goodman test (F710.690: *Z* = 2.07, *p* = 0.04; F1010.940: *Z* = 2.47, *p* = 0.01; F710.690-F1010.940-: *Z* = 2.45, *p* = 0.01). The mediator variable’s value accounted for (VAF) 84.5, 71.4, and 71% of the variance when the F710.690, F1010.940, and F710.690-F1010.940 variables were used respectively.

**Figure 3:**
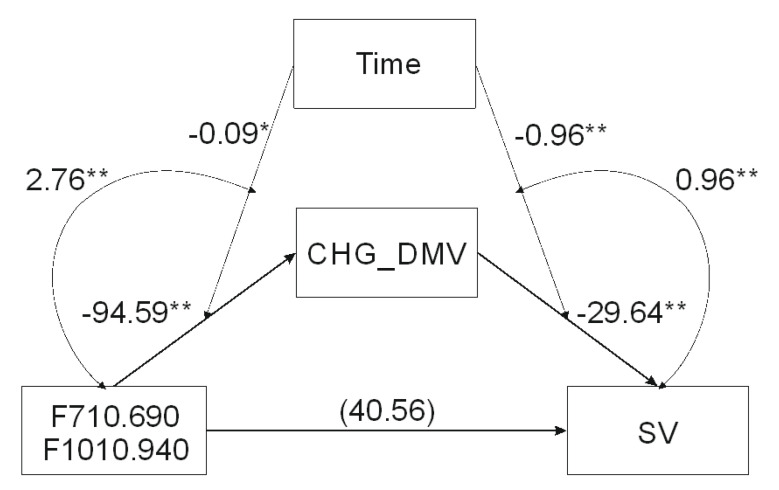
Mediation analysis of the compounds associated with infrared spectral regions of 710–690 and 1010–940 cm^−1^ to demonstrate their effect on sequence variation (SV) via demethylation of the CHG sequence context (CHG_DMV) moderated by time of tissue culture. * *p* < 0.05, ** *p* < 0.01.

**Figure 4:**
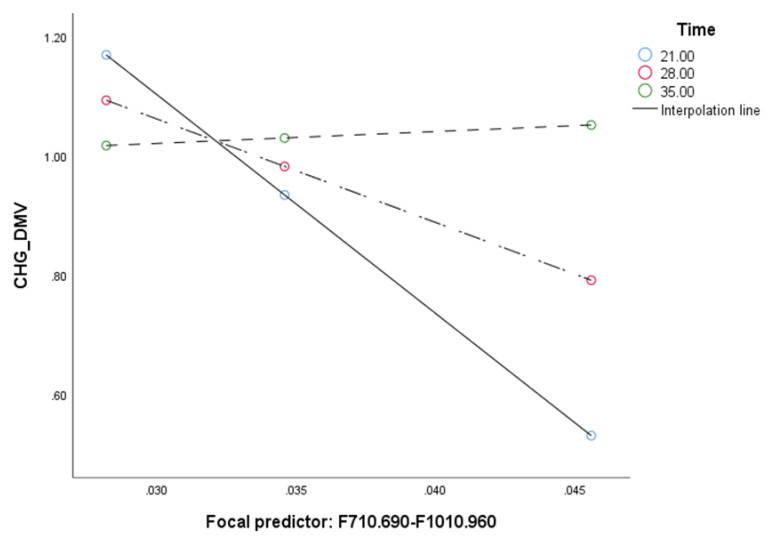
Conditional effect of the integrated absorbance area for regions 710–690 and 1010–940 cm^−1^ (F710.690-F1010.940; predictor) under different durations of in vitro anther culture.

**Figure 5:**
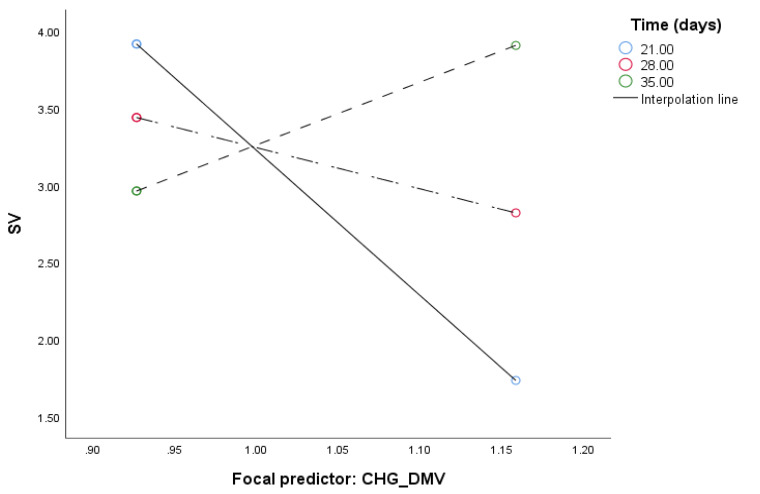
Conditional effect of demethylation of the CHG sequence context (CHG_DMV; predictor) under different durations of in vitro anther culture.

When the analysis tested relationship between F710.690-F1010.940 and SV mediated by CHG_DMV and moderated by the time of in vitro tissue cultures (Table 3) the model (Figure 3) was significant (*F*(4, 30) = 66.25, *p* < 0.0001) and explained 90% of the variance. The interaction between F710.690-F1010.940 and time as well as between CHG_DMV and time was significant based on bootstrap confidence intervals, indicating that mediation was moderated by time. Test of the highest order of unconditional interaction between F710.690-F1010.940 and time (*F*(1, 31) = 9.89, *R*^2^_chang_ = 0.1848, *p* < 0.01) as well as between CHG_DMV and SV (F(1, 30) = 28.32, *R^2^_chang_* = 0.096, *p* < 0.0001) were also significant and explained 18.48% and 9.6% of variance, respectively. Johnson–Neuman’s statistics indicated that moderation of F710.690-F1010.940 by time was significant through 30 days (Figure 4), and CHG_DMV through 28 days of in vitro tissue culture (Figure 5; data not shown). The indirect effect of moderated mediation was significant through 21 days of in vitro culture (Table 3). The indirect and direct effects of the moderated mediation calculated as β*a* × β*b* and β*a* × (β*b* + *c*’) equaled 2804.153 and 2844.715, respectively. Thus, the VAF values of the mediator variables accounted for 98.57% of the variance. The significance of the moderated mediator was also confirmed by the Goodman test (*Z* = 3.29, *p* < 0.001).

## 3. Discussion

To identify compounds that function as predictors in mediation analysis and explain the role of CHG_DMV in SV induced by in vitro tissue culture, we searched the biochemical profiles of leaves of barley plants exposed to variable growth conditions. We used ATR-FTIR spectroscopy to perform a comprehensive analysis of all major compounds present in barley leaves.

We decided to scan the entire mid-infrared spectrum to identify regions that potentially contributed to SV in plants regenerated by an in vitro anther culture. We predicted that this approach would allow us to verify the putative involvement of some vital biochemical pathways, including the methionine cycle [53] and its crucial compound S-adenosyl-L-methionine (SAM), in DNA methylation [54], which could contribute to SV [19]. Moreover, we suspected that compounds indicative of stress caused by in vitro culture, such as lipids [55], polyamines [56], and polysaccharides [57], might also affect DNA methylation.

Mediation analysis of the fragmented ATR-FTIR spectrum demonstrated that the wavenumber ranges 710–690 and 1010–940 cm^−1^ contributed significantly to performance of the statistical model if the integrated absorbances were treated as independent variables. When the two wavenumber ranges were combined into a single variable, the mediation was also significant, explaining as much as 71% of the variance. The VAF value was < 80%, which clearly indicates that factors other than the moderator (CHG_DMV) would need to be incorporated into the model to fully explain SV induced by an in vitro anther culture. Previously, we found that the process of green plant regeneration was moderated by the presence of Cu^2+^ and Ag^+^ ions in the medium, and depended on the time duration of the in vitro anther culture [58] (under review). Acquisition of plants via in vitro cultures can occur from explants by direct organogenesis or embryogenesis without previously formed callus [59,60] or vicariously, when new shoots or embryos occurred on already formed callus (indirect organogenesis or embryogenesis) [61]. It is being suggested that under tissue culture conditions direct embryogenesis may proceed an indirect one. Thus, the role of time could be related to a moment when plant regeneration via indirect exceeds direct embryogenesis what might be linked to the utilization of β-glucans as a source of glucose under darkness [62]. Moreover, it was also demonstrated by us [38] that time was influential in the case of mediation between DNA demethylation of the CG sites and SV due to the presence of copper and silver ions in the medium. In this study, the time duration of in vitro culture was also a significant moderator. When these elements were incorporated into the model of moderated mediation, the VAF value increased to nearly 100%, indicating that most of the essential factors were included in the model.

The 710–690 cm^−1^ band region is putatively associated with cellulose, whereas another band at approximately 665 cm^−1^ was also detected in the spectra of the examined material [47,48]. However, mediation analysis did not show a significant effect of the 665 cm^−1^ band, possibly because of the presence of cellulose in dissimilar forms and/or at different crystallinity levels, generating separate peaks. This contradiction may result from diverse patterns of intermolecular bonds and high sensitivity of the O-H out-of-plane bending vibrations due to hydrogen bonding. Variation in the position of the peak attributed to cellulose, independent of the hydrogen bonding pattern and crystallinity level, has been reported previously [47,48]. The band region at 700 cm^−1^ may be associated with a more amorphic fraction of cellulose compared with that at 665 cm^−1^, and consequently only this fraction may be relevant for mediation analysis. Abiotic stresses during the regeneration process may be more influential on the amorphic fraction of cellulose than on the crystalline fraction. It is possible that stress due to the supply of Cu^2+^ and Ag^+^ ions in the regeneration medium affected the status of cellulose primarily in a relatively more amorphic phase. In *Miscanthus giganteum*, a slight shift in the cellulose peak in this region of frequency has been observed as a result of mild chilling stress [63], which was associated with the formation of a more compact cellulose structure. The band at 700 cm^−1^ may be assigned not only to cellulose but also to the C-S stretching vibrations, which may be attributed to thiol or thioester functional groups. In the context of the apparent involvement of spectral data in the methylation process, an indication of SAM as a compound associated with this spectral region seems to be the most rational. The C-S stretch vibrations in the spectral region displayed a relatively strong signal, and the integrated absorbance may well explain the observed band even at a reasonably low concentration of the compound. Concerning the 710–690 cm^−1^ band region it should be stressed that mediation was not significant based on the indirect effect and nearly significant based on the completely unstandardized indirect effect. We tend to think that such a result is explained by a relatively small sample size rather than by a lack of mediation. The notion is supported by the fact that the compounds reflecting the 710–690 cm^−1^ spectrum region are documented in processes that may affect sequence variation.

Other spectra that acted as predictors in the mediation model included absorbances between 1010 and 940 cm^−1^. This spectral range constitutes a shoulder of intricate broadband, with a maximum at 1058 cm^−1^. The deconvolution of the band region generated two main components (located at 1069 and 1036 cm^−1^) and two much smaller peaks (at 991 and 960 cm^−1^), which could modulate the data associated with the F1010.940 variable. The broad peak with a maximum at 1058 cm^−1^ is greatly influenced by cellulose and hemicellulose [42]. In the hemicellulose fraction, β-glucans seemed to have the most substantial effect as signals, as pectins and other putative polysaccharides did not show clear specific peaks.

Since the contribution of β-glucans to the band region is marked, a similarly essential effect may be attributed to β-glucans in the 1010–940 cm^−1^ region. Therefore, both β-glucans and cellulose may contribute to mediation, and variability in the proportion of contribution between these two compounds may affect the model in mediation analysis.

The ATR-FTIR analysis of the 710–690 and 1010–940 cm^−1^ spectrum ranges suggests that these regions are associated with both cellulose and β-glucans, but the assignment of the 710–690 bands to cellulose was much more apparent than to β-glucans. Despite that the 710–690 cm^−1^ band is very weak, we are convinced that it reflects biological differentiation, due to both plant individual variability and the applied treatments. The technical repetitions (data not shown) were almost identical and did not infer observed data. We also exclude the randomness effect because the used spectrometer provided the spectra with the signal-to-noise ratio above 35,000:1. The absorbance in the 1010–940 cm^−1^ range showed an equal contribution of β-glucans and cellulose, although it was difficult to split the share. Thus, both β-glucans and cellulose can be considered as mediators, but the variability in the glucan content may be crucial for the model.

At a first glance, the role of β-glucans as a putative predictor of mediation seems suspicious. However, studies on cell wall signaling suggest that mitochondria and the cell wall cooperate to mediate stress responses [64]. Stresses can also affect the rate of respiratory electron transport, and mitochondria act as hubs for signaling to other cellular organelles [65]. β-glucans are a major component of the cell wall [66]. In vitro anther cultures are maintained in the dark [25]. Under such conditions, β-glucans are a source of metabolizable glucose [67]. Glucose from the cell wall in the form of (1,3;1,4)-β-glucans is mobilized quicker than glucose from starch (which is not available in tissue culture carried out in the dark). The process of β-glucan depolymerization requires only two enzymes: (1,3;1,4)-β-glucan endohydrolase and the exo-acting β-glucan glucohydrolase [68], biosynthesis of lipids [69] that may act as signaling compounds [70,71] affecting gene expression [72,73]. Interestingly, β-1,3-linked D-glucose in the cell wall also protects plants from external stresses [74]. Other pathways linked to glycolysis and the tricarboxylic acid cycle (TCA) include the methionine cycle and ethylene biosynthesis pathway.

It is known that the ethylene biosynthesis pathway involving SAM [75,76,77] contributes to DNA methylation [78]. At low SAM concentration, the expression of genes involved in DNA repair and lipid and glucose metabolism is altered [79]. The decarboxylated form of SAM (dcAdoMet) is reported to block cytosine methylation [80]. Decarboxylated SAM may block DNA methylation by SAM and is also involved in polyamine synthesis [80]. Polyamines participate in the regulation of transcription, translation, cell growth, and apoptosis [80]. Thus, either changes in the expression of genes involved in DNA repair or the presence of mutations (such as C→T transitions) not recognized by DNA repair systems may act as a link between DNA methylation and SV induced by in vitro tissue culture [81]. Alternatively, SAM-e could be cleaved reductively by an iron-sulfur cluster-containing enzymes, producing an intermediate called 5′-deoxyadenosyl radical [82]. Such radicals may modify tRNA [83], causing rearrangements and other post-transcriptional modifications [84]. The 5′-deoxyadenosyl radical removes a hydrogen from proteins, RNA, or DNA (substrate) initiating a radical mechanism [80]. Radicals may induce DNA modifications leading to DNA mutations [85]. SAM is a key player in the methionine cycle, leading to ethylene biosynthesis. The level of ethylene is dependent on light conditions, and its regulation is dependent on enzymes participating in the ethylene pathway [86]. Since ethylene is a phytohormone that may affect gene expression and may depend on light conditions it may potentially contribute to SV. Thus, SAM and its derivatives may play a role in DNA mutations.

In the ATR-FTIR spectrum, the 1010–940 cm^−1^ range was linked to cellulose and β-glucans. Mediation analysis of the 1010–940 cm^−1^ range explained less variance, and mediation was not classified as full. This suggests that either cellulose is less critical for mediation and only influences the signal from β-glucans, or that cellulose participates in different mechanisms affecting SV. One possibility is that the level of cellulose affects Cu^2+^ and Ag^+^ ions present in the regeneration medium and delivered to the cell via the cell wall. If this is true, then insufficient concentration of Cu^2+^ and Ag+ ions in the medium should alter the biochemical pathways that require these ions. However, in this study, Cu^2+^ and Ag^+^ ions were not incorporated in the model tested, as they were statistically insignificant (data not shown). The other possibility concerns the putative signaling pathway that links cellulose and mitochondria. Two pentatricopeptide repeat proteins, cell wall maintainer1 (cwm1) and cwm2, are involved in editing mitochondrial transcripts that encode subunits of the respiratory complex III, which is linked to the maintenance of cell wall integrity under stress and activation of retrograde mitochondrial signaling via ANAC017, a transcription factor involved in retrograde signaling to the nucleus upon mitochondrial dysfunction [87]. A complex hierarchy of transcription factors exists downstream of ANAC017, including ANAC and WRKY transcription factors associated with organellar signaling and senescence, and ethylene- and gibberellic acid-related transcription factors involved in stress responses [88].

Recently, a study showed that mitochondrial nicotinamide adenine dinucleotide (NAD) reduced oxidation links the TCA with methionine metabolism and nuclear DNA methylation [89]. The methionine cycle is a vital pathway in DNA methylation [90]. Studies show that 1-aminocyclopropane-1-carboxylic acid (ACC), the immediate precursor of ethylene, is involved in the regulation of the cell wall function and response to cell wall damage [91]. Thus, we believe that the signal from cellulose coincides with that from β-glucans using, at least to some extent, the same pathways that lead to DNA methylation.

Analysis of the moderated mediation showed that the model was highly influential, and inclusion of the time duration factor in the in vitro culture resulted in 98.57% VAF. Thus, the model explained nearly all variance. This is not surprising, as many cellular processes, including reprogramming, depend on time. The moderation effect of time was also observed when analyzing moderated moderation, where the moderation was conditional on the time of the entire in vitro culture period (21–30.1 days when relations between β-glucans and CHG_DMV were assumed, and until 28 days when CHG_DMV and SV were analyzed). Previously, we proposed that the moment when β-glucans become the predominant source of glucose in the cell coincides with the transition from direct to indirect embryogenesis [38]. However, in this study, the time of in vitro tissue culture was a non-significant moderator, possibly because of a different reason. It is possible that after 28 days of tissue culture, demethylation of the CHG context results in such a low level of methylated cytosines that the cell repair systems can easily cope with putative mutagenic modifications of cytosines. If this hypothesis is correct, the level of mutations after 28 days of tissue culture should be stabilized. Our results may also be interpreted in terms of the cell reprogramming process [12] affecting DNA demethylation change. Further studies are needed to confirm or reject this hypothesis.

Finally, it should be emphasized that the moderated mediation model presented in this study failed to explain the demethylation of CG sites, although the reason for this result is not apparent. However, we interpret the results in terms of how the process of DNA demethylation and its maintenance differ between the two sequence contexts [7,92]. Epigenetic mechanisms involved in demethylation of the CHG context [93] or maintenance of methylation [94] are most likely crucial here. We cannot exclude the possibility that differences between the two contexts are due to the fact that CG is predominant in euchromatic regions, whereas CHG and CHH sequences are more abundant in heterochromatic regions, and distinct mechanisms regulate the maintenance of the methylation of the two contexts [7].

## 4. Materials and Methods

### 4.1. Plant Material

The experiment was carried out on barley (*Hordeum vulgare* L.) of the NAD2 spring genotype obtained from Poznan Plant Breeding Ltd. (Nagradowice, Poland). The presented research was conducted on donor plants and their regenerants. The donor plants were the generative offspring of regenerants obtained by androgenesis in anther cultures. To obtain donor plants, seeds of the NAD2 genotype were sown and grown under controlled conditions: 16 h light/8 h dark photoperiod, 16 °C/12 °C during day/night, and approximately 190 µE m^−2^ s^−1^ light intensity using sodium lamps. Spikes from these plants were harvested when microspores were in the mid- to the late-uninucleate stage. To induce androgenesis, the spikes were kept in the dark at 4 °C for 21 days. After this time, the anthers were removed and placed on the N6L induction medium containing macro- and microelements [95] supplemented with 2 mg L^−1^ 2,4-dichlorophenoxyacetic acid, 0.5 mg L^−1^ naphthaleneacetic acid, and 0.5 mg L^−1^ kinetin. The anther plates prepared in this way were incubated in the dark at 26 °C. First androgenic structures were transferred onto regeneration medium K4NB [96] supplemented with 0.225 mg L^−1^ 6-benzylaminopurine. The incubation of the callus on the regeneration medium was carried out at temperature 26 °C under the following photoperiod: 16 h light/8 h dark. Appearing green regenerants were transferred to flasks on the N6I rooting medium [95] supplemented with 2 mg L^−1^ indole-3-acetic acid and then to pots and grown and greenhouses. The randomly chosen spikes of doubled haploid regenerants were self-pollinated, and finally, seeds were collected. Twenty four offspring obtained from doubled haploids were grown as donor plants to get regenerants for this experiment. The acquisition of regenerants for the present investigation was carried out according to the procedure described above for the donor plants. The difference was the use of nine variants of induction media, the base composition of which was the same. Still, the supplementation of copper and silver ions and the incubation time differed. Detailed supplementation of the induction media is included in Table 4. Each variant was treated as separate in vitro culture conditions and described as trial M1–M9. Trail M1, in which there are no Ag ions, and Cu ions are at the standard medium level, is a control sample, while trial M2–M9 are test samples. From the pool of 24 donor plants and their regenerants, it was possible to obtain regenerants in all the tested trials (M1–M9) for only one donor plant. This donor plant and its regenerants were taken for analysis. Hence, nine media with different concentrations of CuSO_4_ and AgNO_3_ and the different in vitro tissue culture duration were tested in terms of the influence of these conditions on the efficiency of green regenerant production and observed genetic/epigenetic variability and the biochemical compounds that may affect DNA methylation patterns and sequence variation.

### 4.2. DNA Isolation and Analysis

DNA was extracted from the fresh leaves of a donor plant and its regenerants using the DNeasy MiniPrep Kit (Qiagen, Hilden, Germany). MetAFLP was performed as described previously [25,28]. Quantitative metAFLP characteristics of demethylated CG and CHG sequences were evaluated as described previously [25].

To conduct ATR-FTIR spectroscopy, leaves were collected from barley regenerants and lyophilized using a speed-vac. The lyophilized leaves were homogenized to a fine powder using an agate mortar and pestle, and immediately used for ATR-FTIR analysis. Mid-infrared spectra were acquired using the iZ10 FTIR module of the Nicolet iN10 MX infrared microscope (Thermo Nicolet Corporation, Madison, WI, USA), equipped with a deuterated triglycine sulfate detector and potassium bromide (KBr) beam splitter. Measurements were recorded in the ATR mode using an ATR accessory (Smart Orbit, Thermo Fisher Scientific) equipped with a single-bounce diamond crystal. A pinch of the homogenized tissue was deposited on the crystal and pressurized against its surface using an attached pressure clamp. Sixty-four interferograms were collected and co-added before Fourier transformation at a wavelength ranging from 4000 to 400 cm^−1^ and at a resolution of 4 cm^−1^. The ATR crystal was carefully cleaned using ethanol before each measurement to remove any residual trace of the previous sample. The spectra were ATR, water vapor and baseline corrected using the OMNIC software (v. 9, Thermo Fischer Scientific). Spectra area normalization (1800–900 cm^−1^) and band integrated area calculations were performed using ChemoSpec [97], while spectra plots were generated using hyperSpec [98], the packages in R programming language [99]. To resolve overlapping peaks, deconvolution was performed using the Gaussian function and nonlinear least-squares fitting [100]. The whole spectrum was initially divided into sections of 10 cm^−1^, and each fragmented spectrum region was then subjected to a mediation analysis [101].

### 4.3. Mediation Analysis

The mediation analysis was performed in the SPSS software v. 26 (https://www.ibm.com/support/pages/node/874712) using the A. F. Hayes Process v. 3.4 macro [101]. Model number 4 and 59 were exploited. Demethylation (DMV), demethylation of the CG contexts (CG_DMV), and demethylation of the CHG contexts (CHG_DMV) were used as putative mediators of sequence variation (SV). The infrared spectrum range (940–1010) was tested as the putative predictor of mediation. The mediation strength was measured using the variance accounted for (VAF) [102] value of the mediator (VAF = indirect effect (IE)/total effect (TE)), indirect, partial, and complete standardized indirect effects of X on Y [103], and the Goodman test [104].

The minimum population size was calculated using the G-Power software [105]. Squared multiple correlation (*r*^2^) was set to 0.1 to calculate the effect size (*f*^2^) at α = 0.05 with three variables and a fixed value of power (1-*β* error probability = 0.28).

## Figures and Tables

**Table 1:** Quantitative metAFLP characteristics and corresponding infrared spectroscopy data of barley regenerants derived by in vitro tissue culture under nine different trials (M1–M9).

Sample No.	Trial	Independent Variable ^1^			Mediator ^1^			Dependent Variable ^1^	Moderator
		F710.690	F1010.940	F710.690-F1010.940	DMV	CG_DMV	CHG_DMV	SV	Time (Days)
1	M1	0.0012	0.0443	0.0455	1.6210	0.4636	1.1583	3.7058	21
2	M1	0.0021	0.0267	0.0288	1.3900	0.2325	1.1588	2.5488	21
3	M1	0.0018	0.0371	0.0389	1.3900	0.2315	1.1584	2.5479	21
4	M1	0.0014	0.0317	0.0332	1.3900	0.2315	1.1584	2.7794	21
5	M1	0.0011	0.0388	0.0399	1.6210	0.4630	1.1583	3.2423	21
6	M2	0.0010	0.0283	0.0293	1.6220	0.4637	1.1585	2.5481	28
7	M2	0.0015	0.0287	0.0302	1.6220	0.4650	1.1584	2.3171	28
8	M2	0.0018	0.0322	0.0339	1.3900	0.4640	0.9268	2.5482	28
9	M3	0.0014	0.0274	0.0288	1.3900	0.4640	0.9268	2.5482	35
10	M3	0.0014	0.0264	0.0279	1.6210	0.4636	1.1583	3.7058	35
11	M3	0.0008	0.0454	0.0461	1.6210	0.4637	1.1584	3.9377	35
12	M3	0.0018	0.0263	0.0281	1.3900	0.4640	0.9268	2.5482	35
13	M3	0.0019	0.0255	0.0275	1.3830	0.4616	0.9224	4.3802	35
14	M4	0.0011	0.0282	0.0293	1.3900	0.4640	0.9268	2.5482	28
15	M4	0.0013	0.0325	0.0338	1.3900	0.4640	0.9268	2.3166	28
16	M5	0.0010	0.0329	0.0339	1.3900	0.4637	0.9268	2.5482	35
17	M5	0.0009	0.0484	0.0493	1.3900	0.4630	0.9266	3.7055	35
18	M5	0.0009	0.0483	0.0491	1.6220	0.6952	0.9271	3.4748	35
19	M5	0.0018	0.0294	0.0312	1.8530	0.6954	1.1582	3.7057	35
20	M6	0.0011	0.0353	0.0364	0.9270	0.0000	0.9271	2.5487	21
21	M6	0.0013	0.0343	0.0356	1.3900	0.4640	0.9268	2.5482	21
22	M6	0.0016	0.0238	0.0254	0.9270	0.0000	0.9268	2.5484	21
23	M6	0.0018	0.0311	0.0329	1.1580	0.2323	0.9268	2.5483	21
24	M6	0.0015	0.0331	0.0346	1.1580	0.2315	0.9271	2.3167	21
25	M7	0.0014	0.0268	0.0283	1.8530	0.6944	1.1581	4.6315	35
26	M7	0.0009	0.0482	0.0492	1.6210	0.4630	1.1584	2.5478	35
27	M7	0.0010	0.0373	0.0383	1.3900	0.4630	0.9266	3.9370	35
28	M8	0.0009	0.0440	0.0449	0.0000	0.0000	0.0000	13.5169	21
29	M8	0.0004	0.0533	0.0537	0.0000	0.0000	0.0000	13.5170	21
30	M8	0.0014	0.0426	0.0440	0.0000	0.0000	0.0000	14.0089	21
31	M9	0.0013	0.0441	0.0454	2.0840	0.9267	1.1582	4.4006	28
32	M9	0.0015	0.0361	0.0377	1.1580	0.2320	0.9268	2.7798	28
33	M9	0.0013	0.0388	0.0400	1.1580	0.2320	0.9268	2.7798	28
34	M9	0.0016	0.0335	0.0351	1.1580	0.2320	0.9268	2.5486	28
35	M9	0.0019	0.0223	0.0243	2.0 940	0.9320	1.1651	3.4931	28

^1^ DMV, demethylation; CG_DMV, demethylation of the CG context; CHG_DMV, demethylation of the CHG context; SV, sequence variation; F710.690, area under the spectral range from 710 to 690 cm^−1^; F1010.940, area under the spectral range from 1010 to 940 cm^−1^; F710.690-F1010.940, combination of F710.690 and F1010.940.

**Table 2:** Outcomes of mediation analyses from F710.690, F1010.940, and F710.690-F1010.940 to sequence variation assessing indirect effects. The unstandardized coefficients are given and explained in the table.

Model	Effects						95% CI	
	*R* ^2^	*c*’	*a*	*b*	*c*	IE	L	U
F710.690- > CHG_DMV- > SV	0.79	−436.37	274.82 *	−8.59 ***	−2797.49 *	−0.299 ^†^	−0.56 ^†^	0.06 ^†^
F1010.940- > CHG_DMV- > SV	0.80	51.35	−15.57 *	−8.24 ***	179.63 **	128.28	0.18	286.98
F710.690-F1010.940- > CHG_DMV- > SV	0.80	53.64	−15.98 *	−8.24 ***	185.26 ***	131.62	0.18	298.27

F710.690, F1010.940, and F710.690-F1010.940 are the ATR-FTIR; CHG_DMV (mediator) states for DNA demethylation of the CHG contexts evaluated by metAFLP approach; SV is sequence variation also evaluated by the metAFLP approach and shared among anther culture derived regenerants of barley; *c*’ = direct effect of predictor (IDs) on outcome (SV) while controlling for the mediator (CHG_DMV); *a* = effect of the predictor (ID) on the mediator (CHG_DMV); *b* = effect of the mediator (CHG_DMV) on the outcome (SV); *c* = total effect focal predictor (F710.690, F1010.940, and F710.690-F1010.690) on the outcome (SV); IE = indirect effect of predictor (ID) on outcome (SV) through the mediator (CHG_DMV); *R*^2^—the amount of variance explained by the model; * *p* < 0.05, ** *p* < 0.01; *** *p* < 0.001; L and U are lower and upper values of 95% confidence interval (CI). ^†^—complete standardized indirect effect (C IE).

**Table 3:** The outcomes (unstandardized regression coefficients) of mediation analyses from F710.690-F1010.940 to SV assessing the indirect effects of CHG_DMV (indicated by 95% confidence interval) moderated by the time of in vitro tissue culture. For further explanation, see Table 2.

Model: Moderated Mediation	*B*	*SE*	95% CI L	95% CI U
*c’*	40.5613	25.167	−10.8377	91.9603
*a*	−94.5994 ***	25.8835	−147.3903	−41.8085
Time (moderator)	−0.0887 *	0.0335	−0.1571	−0.0204
Moderation: F710.690-F1010.940 × Time	2.7594 **	0.8776	0.9696	4.5493
*b*	−29.6424 ****	4.0666	−37.9477	−21.3371
Time (moderator)	−0.9608 ****	0.1848	−1.3382	−0.5834
Moderation: CHG_DMV × Time	0.9633 ****	0.181	0.5936	1.333
*c*	40.5613	25.167	−10.8377	91.9603
Conditional indirect effect of F710.690-F1010.940 on SV (F710.690-F1010.940 --> CHG_DMV --> SV)				
Time	*B*	*SE*	L	U
21	344.9937	154.151	9.9749	633.6907
28	46.2799	114.4689	−7.6518	113.7094
35	8.0692	53.3305	−43.0736	72.1826

* *p* < 0.05, ** *p* < 0.01; *** *p* < 0.001; **** *p* < 0.0001.

**Table 4:** Concentrations of ions and number of days applied in trials (M1–M9).

Trial	Cu^2+^ (µM)	Ag^+^ (µM)	Time (Days)
M1	0.1	0	21
M2	0.1	10	28
M3	0.1	60	35
M4	5	60	28
M5	5	0	35
M6	5	10	21
M7	10	10	35
M8	10	60	21
M9	10	0	28

## Abbreviations

5 hmC	5-hydroxymethylcytosine
5 mC	5-methylcytosine
ATR-FTIR	Attenuated Total Reflectance Fourier Transform Infrared
CG_DMV	demethylation of the CG contexts
CHG_DMV	demethylation of the CHG contexts
dcAdoMet	decarboxylated form of SAM
DMV	demethylation
IDM	increased DNA methylation complex
IF	indirect effect
metAFLP	methylation-sensitive Amplified Fragment Length Polymorphism
MMR	mismatch repair
NER	nucleotide excision repair
RdDM	RNA-directed DNA methylation
ROS1	REPRESSOR OF SILENCING 1
SAM	S-adenosyl-L-methionine,
SV	sequence variation
TCA	tricarboxylic acid cycle
TE	total effect
VAF	variance accounted for
